# Supplementary material for: Simulations of the effect of diffusion on asymmetric spin echo based quantitative BOLD: An investigation of the origin of deoxygenated blood volume overestimation
Source: Neuroimage. 2019 Nov 1;201:116035. doi: 10.1016/j.neuroimage.2019.116035 (PMC6996000; doi:10.1016/j.neuroimage.2019.116035)
Supplement: Multimedia component 1 [file mmc1.pdf]

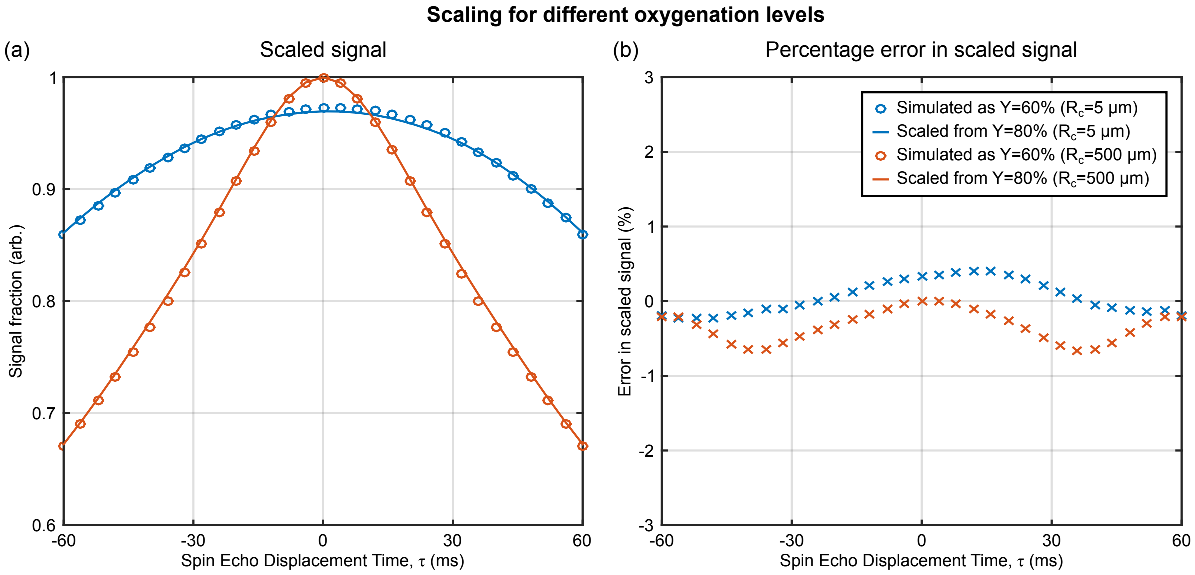


**Fig. S1.** Monte Carlo simulations can be accelerated to calculate different oxygenations by saving the phase accumulated by each proton for a nominal oxygenation value. Since phase scales linearly with oxygenation, the simulated phase accrual can be scaled to a target oxygenation prior to the estimation of the signal (Blockley et al., 2008). (a) Two examples are shown here: (i) a vessel radius of 5 μm simulated as Y=60% (blue markers) and simulated as Y=80% and scaled to Y=60% (blue line) and (ii) a vessel radius of 500 μm simulated as Y=60% (red markers) and simulated as Y=80% and scaled to Y=60% (red line). (b) The percentage error between the simulated and scaled signals are also displayed i.e. $\left(S^{simulated} - S^{scaled}\right)/S^{simulated}$.

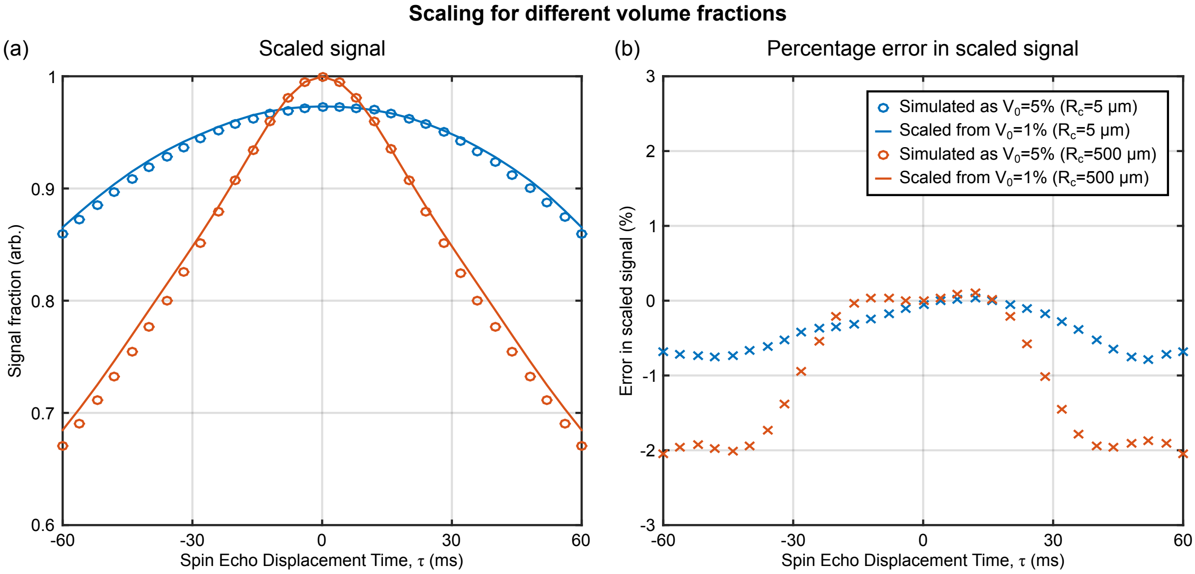


**Fig. S2.** Monte Carlo simulations can also be accelerated to calculate different blood volume fractions. This is achieved by estimating an oxygenation and vessel radius dependent shape function (Dickson et al., 2011; Kiselev and Posse, 1999). This shape function can then be arbitrarily scaled for different blood volume fractions. (a) Two examples are shown here: (i) a vessel radius of 5 μm simulated as $V_0$=5% (blue markers) and simulated as $V_0$=1% and scaled to $V_0$=5% (blue line) and (ii) a vessel radius of 500 μm simulated as $V_0$=5% (red markers) and simulated as $V_0$=1% and scaled to $V_0$=5% (red line). (b) The percentage error between the simulated and scaled signals are also displayed i.e. $\left(S^{simulated} - S^{scaled}\right)/S^{simulated}$.

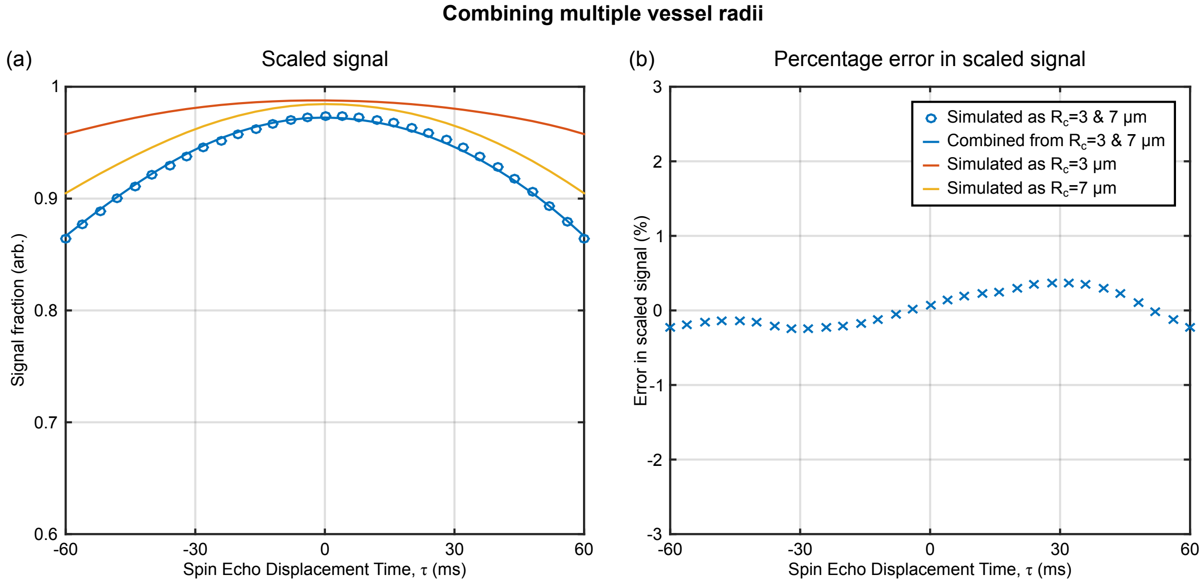


**Fig. S3.** The simulation of systems with multiple vessel radii can be simulated by combining the results of multiple single vessel radius simulations (Dickson et al., 2011; Kiselev and Posse, 1999). (a) In this example, simulations of a system with $V_0$=5% equally split between vessels with $R_c$=3 µm and $R_c$=7 µm (blue markers). Single vessel simulations of $R_c$=3 µm (red line) and $R_c$=7 µm (yellow line) with $V_0$=2.5%. The combined effect of these single vessel simulations is obtained by taking the product of these signal curves (blue line). (b) The percentage error between the simulated and combined signals are also displayed i.e. $\left(S^{simulated} - S^{combined}\right)/S^{simulated}$.

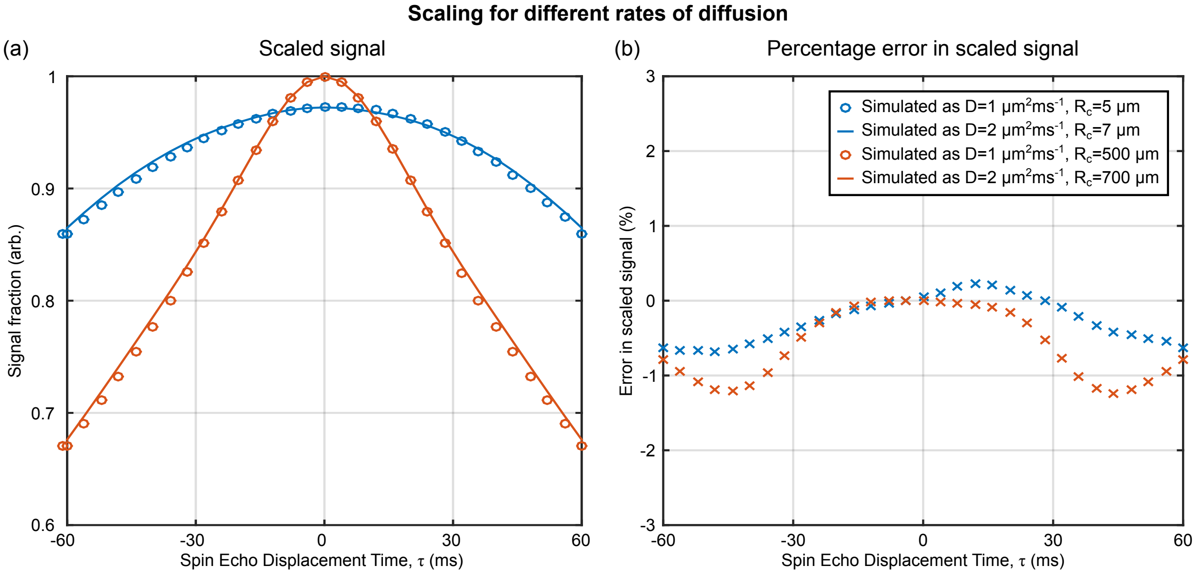


**Fig. S4.** Monte Carlo simulations can also be accelerated to simulate the effect of different diffusion coefficients, D. The effect of diffusion is a function of vessel radius and diffusion coefficient through the characteristic diffusion time $\tau_D \propto R_c^2/D$ (Yablonskiy and Haacke, 1994). Therefore, the effect of a change in diffusion can be simulated by scaling the vessel radius e.g. scaling D by a factor of 2 requires $R_c$ to be scaled by $\sqrt{2}$. (a) Two examples are shown here: (i) a vessel radius of 5 μm simulated with D=1 $\mu m^2 ms^{-1}$ (blue markers) compared with a vessel radius of 7 μm simulated with D=2 $\mu m^2 ms^{-1}$ (blue line) and (ii) a vessel radius of 500 μm simulated with D=1 $\mu m^2 ms^{-1}$ (red markers) compared with a vessel radius of 700 μm simulated with D=2 $\mu m^2 ms^{-1}$ (red line). (b) The percentage error between the simulated and scaled signals are also displayed i.e. $\left(S^{simulated} - S^{scaled}\right)/S^{simulated}$.

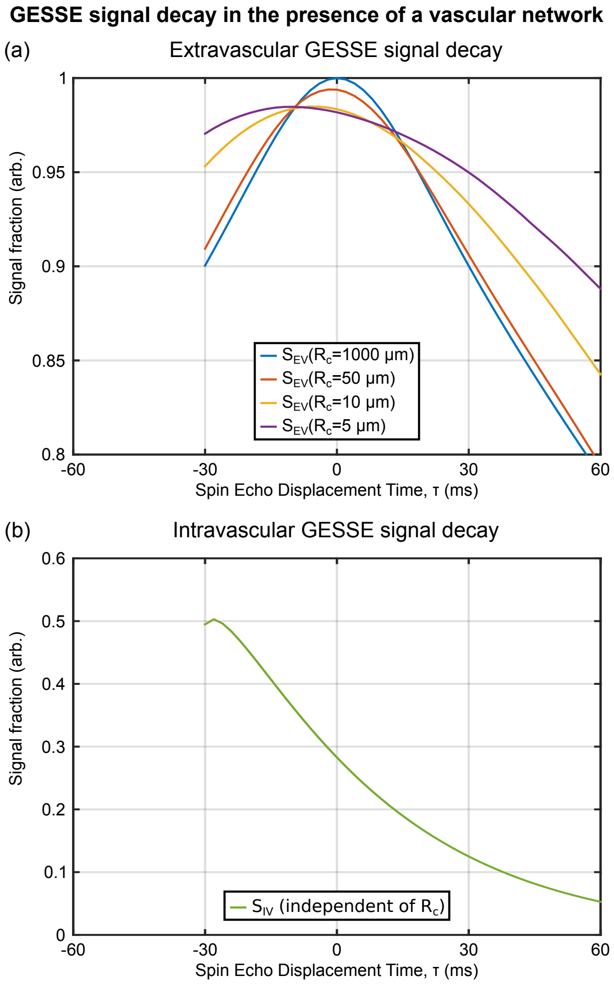


**Fig. S5.** Examples of the signal decay from the GESSE pulse sequence. The extravascular signal ($S_{EV}$) decay is observed to be asymmetric with respect to τ=0 as the vessel radius is reduced. (b) The intravascular signal ($S_{IV}$) decay shows considerable signal attenuation which is highly asymmetric with respect to τ and appears almost exponential in form.

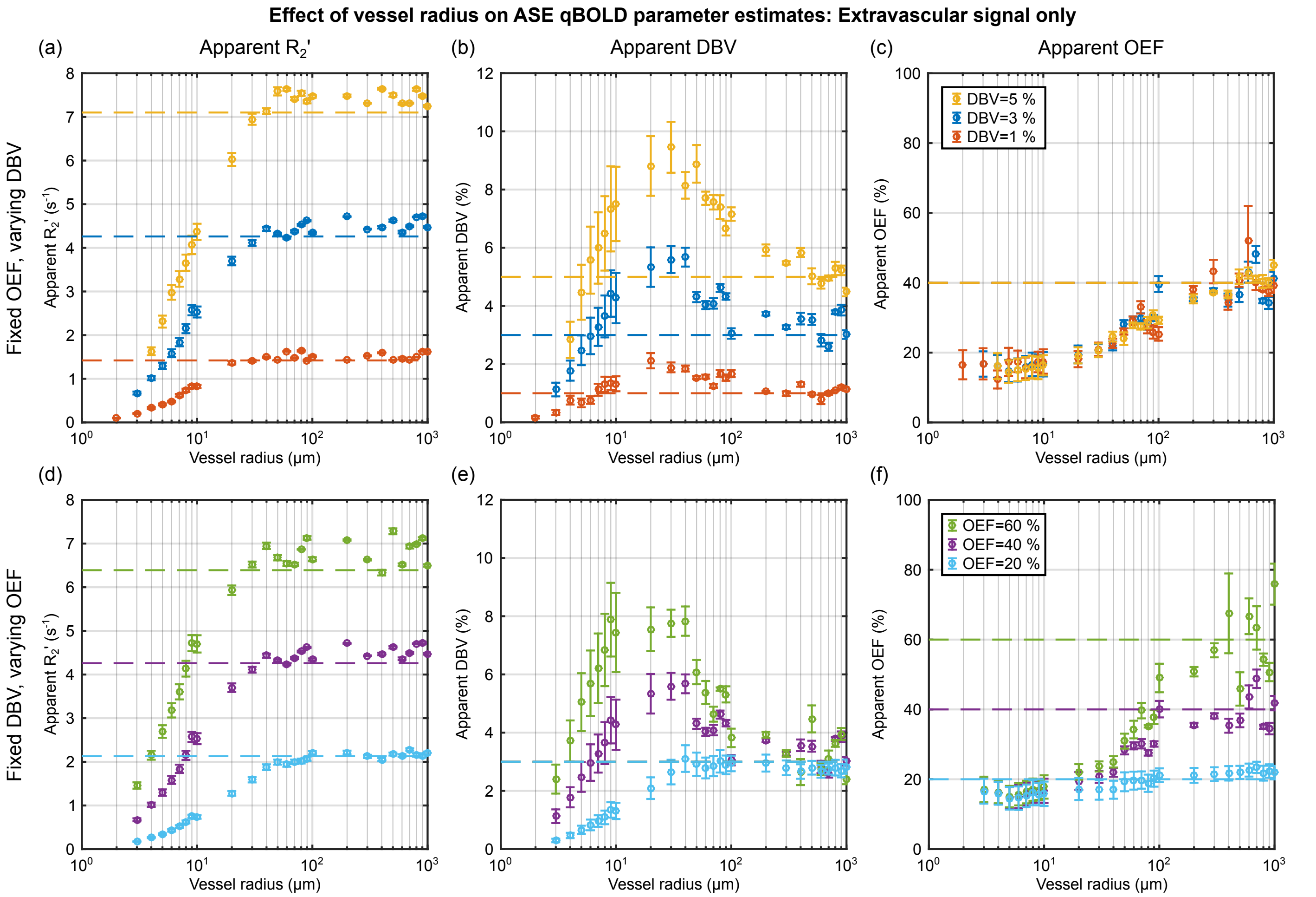


**Fig. S6.** Reproduction of Fig. 3 for simulations of the extravascular signal only. As in Fig. 3, simulations were first performed with a fixed OEF ($E_0$=40%) and three DBV values (top) then with a fixed DBV ($V_0$=3%) and three values of OEF (bottom). The apparent $R_2'$ (left) is estimated for each OEF-DBV pair and presented alongside the $R_2'$ values predicted by the SDR qBOLD model (dashed lines). Likewise the apparent DBV (centre) and apparent OEF (right) are presented alongside the true DBV and OEF, respectively, (dashed lines).

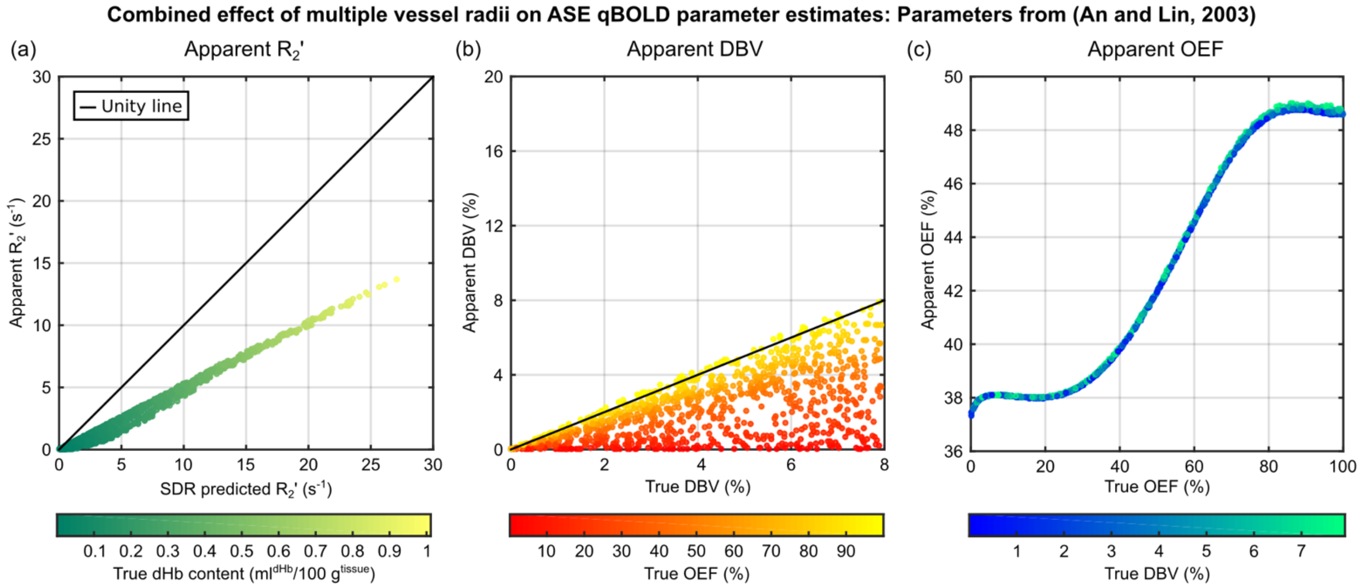


**Fig. S7.** The effect of multiple vessel radii simulations on the qBOLD parameter estimates was considered by generating many pairs of OEF and CBV values. ASE pulse sequence parameters were $t_E$=64 ms with τ=0 and τ=10 to 18 ms in 4 ms steps following the work of (An and Lin, 2003). (a) The apparent $R_2'$ is linearly dependent on the $R_2'$ predicted by the SDR model, in common with the parameters used in Fig. 7 but with a shallower gradient. (b) Uncertainty is similarly observed in the apparent DBV, but with a reduced range of values. (c) In contrast to Fig. 7c, the apparent OEF has a largely monotonic relationship with the true OEF. Markers are coloured to reflect true dHb content, true OEF and true DBV for parts (a), (b) and (c), respectively.

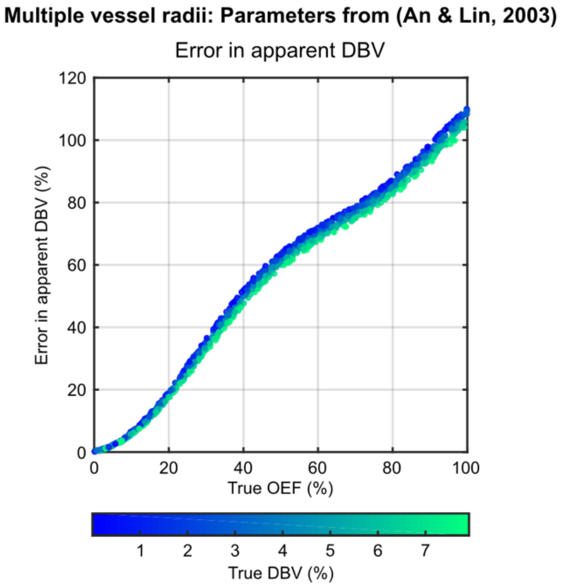


**Fig. S8.** The uncertainty in DBV in Fig. S6 was investigated by plotting apparent DBV as a function of true OEF. ASE pulse sequence parameters are the same as detailed in Fig. S6. The results suggest that the error in the apparent DBV is OEF dependent. Markers are coloured to reflect their true DBV.

## References

An, H., Lin, W., 2003. Impact of intravascular signal on quantitative measures of cerebral oxygen extraction and blood volume under normo- and hypercapnic conditions using an asymmetric spin echo approach. Magn. Reson. Med. 50, 708–716.

Blockley, N.P., Jiang, L., Gardener, A.G., Ludman, C.N., Francis, S.T., Gowland, P.A., 2008. Field strength dependence of R1 and R2* relaxivities of human whole blood to prohance, vasovist, and deoxyhemoglobin. Magn. Reson. Med. 60, 1313–1320.

Dickson, J.D., Ash, T.W.J., Williams, G.B., Sukstanskii, A.L., Ansorge, R.E., Yablonskiy, D.A., 2011. Quantitative phenomenological model of the BOLD contrast mechanism. J. Magn. Reson. 212, 17–25.

Kiselev, V.G., Posse, S., 1999. Analytical model of susceptibility-induced MR signal dephasing: effect of diffusion in a microvascular network. Magn. Reson. Med. 41, 499–509.

Yablonskiy, D.A., Haacke, E.M., 1994. Theory of NMR signal behavior in magnetically inhomogeneous tissues: the static dephasing regime. Magn. Reson. Med. 32, 749–763.
